# On Representations of Divergence Measures and Related Quantities in Exponential Families

**DOI:** 10.3390/e23060726

**Published:** 2021-06-08

**Authors:** Stefan Bedbur, Udo Kamps

**Affiliations:** Institute of Statistics, RWTH Aachen University, 52056 Aachen, Germany; bedbur@isw.rwth-aachen.de

**Keywords:** exponential family, cumulant function, mean value function, divergence measure, distance measure, affinity, 60E05, 62H12, 62F25

## Abstract

Within exponential families, which may consist of multi-parameter and multivariate distributions, a variety of divergence measures, such as the Kullback–Leibler divergence, the Cressie–Read divergence, the Rényi divergence, and the Hellinger metric, can be explicitly expressed in terms of the respective cumulant function and mean value function. Moreover, the same applies to related entropy and affinity measures. We compile representations scattered in the literature and present a unified approach to the derivation in exponential families. As a statistical application, we highlight their use in the construction of confidence regions in a multi-sample setup.

## 1. Introduction

There is a broad literature on divergence and distance measures for probability distributions, e.g., on the Kullback–Leibler divergence, the Cressie–Read divergence, the Rényi divergence, and Phi divergences as a general family, as well as on associated measures of entropy and affinity. For definitions and details, we refer to [1]. These measures have been extensively used in statistical inference. Excellent monographs on this topic were provided by Liese and Vajda [2], Vajda [3], Pardo [1], and Liese and Miescke [4].

Within an exponential family as defined in Section 2, which may consist of multi-parameter and multivariate distributions, several divergence measures and related quantities are seen to have nice explicit representations in terms of the respective cumulant function and mean value function. These representations are contained in different sources. Our focus is on a unifying presentation of main quantities, while not aiming at an exhaustive account. As an application, we derive confidence regions for the parameters of exponential distributions based on different divergences in a simple multi-sample setup.

For the use of the aforementioned measures of divergence, entropy, and affinity, we refer to the textbooks [1,2,3,4] and exemplarily to [5,6,7,8,9,10] for statistical applications, including the construction of test procedures as well as methods based on dual representations of divergences, and to [11] for a classification problem.

## 2. Exponential Families

Let Θ≠∅ be a parameter set, μ be a σ-finite measure on the measurable space (X,B), and $P=\{P_{\vartheta }:\vartheta \in \Theta \}$ be an exponential family (EF) of distributions on (X,B) with μ-density

(1) $$f_{\vartheta }\left(x\right)\;=\;C\left(\vartheta \right)\exp\left\{\sum_{j=1}^{k}Z_{j}\left(\vartheta \right)\;T_{j}\left(x\right)\right\}\;h\left(x\right)\;,\;x\in X\;,$$

of $P_{\vartheta }$ for ϑ∈Θ, where $C,Z_{1},\ldots ,Z_{k}:\Theta \to R$ are real-valued functions on Θ and $h,T_{1},\ldots ,T_{k}:\left(X,B\right)\to \left(R^{1},B^{1}\right)$ are real-valued Borel-measurable functions with h≥0. Usually, μ is either the counting measure on the power set of X (for a family of discrete distributions) or the Lebesgue measure on the Borel sets of X (in the continuous case). Without loss of generality and for a simple notation, we assume that h>0 (the set {x∈X:h(x)=0} is a null set for all P∈P). Let ν denote the σ-finite measure with μ-density *h*.

We assume that representation (1) is minimal in the sense that the number *k* of summands in the exponent cannot be reduced. This property is equivalent to $Z_{1},\ldots ,Z_{k}$ being affinely independent mappings and $T_{1},\ldots ,T_{k}$ being ν-affinely independent mappings; see, e.g., [12] (Cor. 8.1). Here, ν-affine independence means affine independence on the complement of every null set of ν.

To obtain simple formulas for divergence measures in the following section, it is convenient to use the natural parameter space

$$\Xi^{*}\;=\;\left\{\zeta \in R^{k}:\int e^{\zeta^{t}T}\;h\;d\mu \;<\;\infty \right\}$$

and the (minimal) canonical representation $\left\{P_{\zeta }^{*}:\zeta \in Z\left(\Theta \right)\right\}$ of P with μ-density

(2) $$f_{\zeta }^{*}\left(x\right)\;=\;C^{*}\left(\zeta \right)\;e^{\zeta^{t}T\left(x\right)}\;h\left(x\right)\;,\;x\in X\;,$$

of $P_{\zeta }^{*}$ and normalizing constant $C^{*}\left(\zeta \right)$ for $\zeta ={\left(\zeta_{1},\ldots ,\zeta_{k}\right)}^{t}\in Z\left(\Theta \right)\subset \Xi^{*}$, where $Z={\left(Z_{1},\ldots ,Z_{k}\right)}^{t}$ denotes the (column) vector of the mappings $Z_{1},\ldots ,Z_{k}$ and $T={\left(T_{1},\ldots ,T_{k}\right)}^{t}$ denotes the (column) vector of the statistics $T_{1},\ldots ,T_{k}$. For simplicity, we assume that P is regular, i.e., we have that $Z\left(\Theta \right)=\Xi^{*}$ (P is full) and that $\Xi^{*}$ is open; see [13]. In particular, this guarantees that T is minimal sufficient and complete for P; see, e.g., [14] (pp. 25–27).

The cumulant function

$$\kappa \left(\zeta \right)\;=\;-\ln\left(C^{*}\left(\zeta \right)\right)\;,\;\zeta \in \Xi^{*}\;,$$

associated with P is strictly convex and infinitely often differentiable on the convex set $\Xi^{*}$; see [13] (Theorem 1.13 and Theorem 2.2). It is well-known that the Hessian matrix of κ at ζ coincides with the covariance matrix of T under $P_{\zeta }^{*}$ and that it is also equal to the Fisher information matrix I(ζ) at ζ. Moreover, by introducing the mean value function

(3) $$\pi \left(\zeta \right)\;=\;E_{\zeta }\left[T\right]\;,\;\zeta \in \Xi^{*}\;,$$

we have the useful relation

(4) π=∇κ,

where ∇κ denotes the gradient of κ; see [13] (Cor. 2.3). π is a bijective mapping from $\Xi^{*}$ to the interior of the convex support of $\nu^{T}$, i.e., the closed convex hull of the support of $\nu^{T}$; see [13] (p. 2 and Theorem 3.6).

Finally, note that representation (2) can be rewritten as

(5) $$f_{\zeta }^{*}\left(x\right)\;=\;e^{\zeta^{t}T\left(x\right)-\kappa \left(\zeta \right)}\;h\left(x\right)\;,\;x\in X\;,$$

for $\zeta \in \Xi^{*}$.

## 3. Divergence Measures

Divergence measures may be applied, for instance, to quantify the “disparity” of a distribution to some reference distribution or to measure the “distance” between two distributions within some family in a certain sense. If the distributions in the family are dominated by a σ-finite measure, various divergence measures have been introduced by means of the corresponding densities. In parametric statistical inference, they serve to construct statistical tests or confidence regions for underlying parameters; see, e.g., [1].

**Definition** **1.** *Let F be a set of distributions on (X,B). A mapping D:F×F→R is called a divergence (or divergence measure) if:*

*(i)*  *D(P,Q)≥0 for all P,Q∈F and D(P,Q)=0⇔P=Q (positive definiteness).*

*If additionally*

*(ii)*  *D(P,Q)=D(Q,P) for all P,Q∈F (symmetry) is valid, D is called a distance (or distance measure or semi-metric). If D then moreover meets*
*(iii)*  *$D\left(P_{1},P_{2}\right)\leq D\left(P_{1},Q\right)+D\left(Q,P_{2}\right)\;$ for all $P_{1},P_{2},Q\in F\;$ (triangle inequality), D is said to be a metric.*

Some important examples are the Kullback–Leibler divergence (KL-divergence):

$$D_{KL}\left(P_{1},P_{2}\right)\;=\;\int f_{1}\;\ln\left(\frac{f_{1}}{f_{2}}\right)\;d\mu \;,$$

the Jeffrey distance:

$$D_{J}\left(P_{1},P_{2}\right)=D_{KL}\left(P_{1},P_{2}\right)+D_{KL}\left(P_{2},P_{1}\right)$$

as a symmetrized version, the Rényi divergence:

(6) $$D_{R_{q}}\left(P_{1},P_{2}\right)\;=\;\frac{1}{q(q-1)}\ln\left(\int f_{1}^{q}\;f_{2}^{1-q}\;d\mu \right)\;,\;q\in R\setminus \left\{0,1\right\}\;,$$

along with the related Bhattacharyya distance $D_{B}\left(P_{1},P_{2}\right)=D_{R_{1/2}}\left(P_{1},P_{2}\right)/4$, the Cressie–Read divergence (CR-divergence):

(7) $$D_{CR_{q}}\left(P_{1},P_{2}\right)\;=\;\frac{1}{q(q-1)}\int f_{1}\;\left[{\left(\frac{f_{1}}{f_{2}}\right)}^{q-1}\;-\;1\right]\;d\mu \;,\;q\in R\setminus \left\{0,1\right\}\;,$$

which is the same as the Chernoff α-divergence up to a parameter transformation, the related Matusita distance $D_{M}\left(P_{1},P_{2}\right)=D_{CR_{1/2}}\left(P_{1},P_{2}\right)/2$, and the Hellinger metric:

(8) $$D_{H}\left(P_{1},P_{2}\right)\;=\;{\left[\int {\left(\sqrt{f_{1}}\;-\;\sqrt{f_{2}}\right)}^{2}\;d\mu \right]}^{1/2}$$

for distributions $P_{1},P_{2}\in F$ with μ-densities $f_{1},f_{2}$, provided that the integrals are well-defined and finite.

$D_{KL}$, $D_{R_{q}}$, and $D_{CR_{q}}$ for q∈R∖{0,1} are divergences, and $D_{J}$, $D_{R_{1/2}}$, $D_{B}$, $D_{CR_{1/2}}$, and $D_{M}\;\left(=D_{H}^{2}\right)$, since they moreover satisfy symmetry, are distances on F×F. $D_{H}$ is known to be a metric on F×F.

In parametric models, it is convenient to use the parameters as arguments and briefly write, e.g.,

$$D_{KL}\left(\vartheta_{1},\vartheta_{2}\right)\;\mathrm{for}\;D_{KL}\left(P_{\vartheta_{1}},P_{\vartheta_{2}}\right)\;,\;\vartheta_{1},\vartheta_{2}\in \Theta \;,$$

if the parameter ϑ∈Θ is identifiable, i.e., if the mapping $\vartheta \mapsto P_{\vartheta }$ is one-to-one on Θ. This property is met for the EF P in Section 2 with minimal canonical representation (5); see, e.g., [13] (Theorem 1.13(iv)).

It is known from different sources in the literature that the EF structure admits simple formulas for the above divergence measures in terms of the corresponding cumulant function and/or mean value function. For the KL-divergence, we refer to [15] (Cor. 3.2) and [13] (pp. 174–178), and for the Jeffrey distance also to [16].

**Theorem** **1.** *Let P be as in Section 2 with minimal canonical representation (5). Then, for $\zeta ,\eta \in \Xi^{*}$, we have*

(9) $$\begin{array}{ccc} D_{KL}\left(\zeta ,\eta \right)\; & = & \;\kappa \left(\eta \right)\;-\;\kappa \left(\zeta \right)\;+\;{\left(\zeta \;-\;\eta \right)}^{t}\;\pi \left(\zeta \right)\\ and\;D_{J}\left(\zeta ,\eta \right)\; & = & \;{\left(\zeta \;-\;\eta \right)}^{t}\;\left(\pi \left(\zeta \right)\;-\;\pi \left(\eta \right)\right)\;. \end{array}$$

**Proof.**  By using Formulas (3) and (5), we obtain for $\zeta ,\eta \in \Xi^{*}$ that

$$\begin{array}{ccc} D_{KL}\left(\zeta ,\eta \right)\; & = & \;\int \left[\ln\left(f_{\zeta }^{*}\right)\;-\;\ln\left(f_{\eta }^{*}\right)\right]\;f_{\zeta }^{*}\;d\mu \\ \; & = & \;\int \left[{\left(\zeta -\eta \right)}^{t}T\;-\;\kappa \left(\zeta \right)+\kappa \left(\eta \right)\right]\;f_{\zeta }^{*}\;d\mu \\ \; & = & \;\kappa \left(\eta \right)\;-\;\kappa \left(\zeta \right)\;+\;{\left(\zeta \;-\;\eta \right)}^{t}\;\pi \left(\zeta \right)\;. \end{array}$$

From this, the representation of $D_{J}$ is obvious. □

As a consequence of Theorem 1, $D_{KL}$ and $D_{J}$ are infinitely often differentiable on $\Xi^{*}\times \Xi^{*}$, and the derivatives are easily obtained by making use of the EF properties. For example, by using Formula (4), we find $\nabla D_{KL}\left(\zeta ,\cdot \right)=\pi \left(\cdot \right)-\pi \left(\zeta \right)$ and that the Hessian matrix of $D_{KL}\left(\zeta ,\cdot \right)$ at η is the Fisher information matrix I(η), where $\zeta \in \Xi^{*}$ is considered to be fixed.

Moreover, we obtain from Theorem 1 that the reverse KL-divergence $D_{KL}^{*}\left(\zeta ,\eta \right)=D_{KL}\left(\eta ,\zeta \right)$ for $\zeta ,\eta \in \Xi^{*}$ is nothing but the Bregman divergence associated with the cumulant function κ; see, e.g., [1,11,17]. As an obvious consequence of Theorem 1, other symmetrizations of the KL-divergence may be expressed in terms of κ and π as well, such as the so-called resistor-average distance (cf. [18])

(10) $$\begin{array}{ccc} D_{RA}\left(\zeta ,\eta \right)\; & = & \;2\;{\left(\frac{1}{D_{KL}\left(\zeta ,\eta \right)}+\frac{1}{D_{KL}\left(\eta ,\zeta \right)}\right)}^{-1}\\ \; & = & \;\frac{2\;D_{KL}\left(\zeta ,\eta \right)\;D_{KL}\left(\eta ,\zeta \right)}{D_{J}\left(\zeta ,\eta \right)}\;,\;\zeta ,\eta \in \Xi^{*}\;,\;\zeta \neq \eta \;, \end{array}$$

with $D_{RA}\left(\zeta ,\zeta \right)=0$, $\zeta \in \Xi^{*}$, or the distance

(11) $$D_{GA}\left(\zeta ,\eta \right)\;=\;{\left[D_{KL}\left(\zeta ,\eta \right)\;D_{KL}\left(\eta ,\zeta \right)\right]}^{1/2}\;,\;\zeta ,\eta \in \Xi^{*}\;,$$

obtained by taking the harmonic and geometric mean of $D_{KL}$ and $D_{KL}^{*}$; see [19].

**Remark** **1.** *Formula (9) can be used to derive the test statistic*

$$\Lambda \left(x\right)\;=\;-2\ln\left(\frac{\sup_{\zeta \in \Xi_{0}}f_{\zeta }^{*}\left(x\right)}{\sup_{\zeta \in \Xi^{*}}f_{\zeta }^{*}\left(x\right)}\right)\;,\;x\in X\;,$$

*of the likelihood-ratio test for the test problem*

$$H_{0}:\;\zeta \in \Xi_{0}\;against\;H_{1}:\;\zeta \in \Xi^{*}\setminus \Xi_{0}\;,$$

*where $\emptyset \neq \Xi_{0}⊊\Xi^{*}$. If the maximum likelihood estimators (MLEs) $\hat{\zeta }=\hat{\zeta }\left(x\right)$ and ${\hat{\zeta }}_{0}={\hat{\zeta }}_{0}\left(x\right)$ of ζ in $\Xi^{*}$ and $\Xi_{0}$ (based on x) both exist, we have:*

$$\begin{array}{ccc} \Lambda \; & = & \;2\;\left[\ln\left(f_{\hat{\zeta }}^{*}\right)-\ln\left(f_{{\hat{\zeta }}_{0}}^{*}\right)\right]\\ \; & = & \;2\;\left[\kappa \left({\hat{\zeta }}_{0}\right)-\kappa \left(\hat{\zeta }\right)+{\left(\hat{\zeta }-{\hat{\zeta }}_{0}\right)}^{t}\;T\right]\\ \; & = & \;2\;D_{KL}\left(\hat{\zeta },{\hat{\zeta }}_{0}\right) \end{array}$$

*by using that the unrestricted MLE fulfils $\pi (\hat{\zeta })=T$; see, e.g., [12] (p. 190) and [13] (Theorem 5.5). In particular, when testing a simple null hypothesis with $\Xi_{0}=\left\{\eta \right\}$ for some fixed $\eta \in \Xi^{*}$, we have $\Lambda =2D_{KL}\left(\hat{\zeta },\eta \right)$.*

Convenient representations within EFs of the divergences in Formulas (6)–(8) can also be found in the literature; we refer to [2] (Prop. 2.22) for $D_{R_{q}}$, $D_{H}$, and $D_{M}$, to [20] for $D_{B}$, and to [9] for $D_{R_{q}}$. The formulas may all be obtained by computing the quantity

(12) $$A_{q}\left(P_{1},P_{2}\right)\;=\;\int f_{1}^{q}\;f_{2}^{1-q}\;d\mu \;,\;q\in R\setminus \left\{0,1\right\}\;.$$

For q∈(0,1), we have the following identity (cf. [21]).

**Lemma** **1.** *Let P be as in Section 2 with minimal canonical representation (5). Then, for $\zeta ,\eta \in \Xi^{*}$ and q∈(0,1), we have:*

$$A_{q}\left(\zeta ,\eta \right)\;=\;\exp\left\{\kappa (q\zeta \;+\;(1-q)\eta )\;-\;[q\kappa (\zeta )\;+\;(1-q)\kappa (\eta )]\right\}\;.$$

**Proof.**  Let $\zeta ,\eta \in \Xi^{*}$ and q∈(0,1). Then,

$$\begin{array}{ccc} A_{q}\left(\zeta ,\eta \right)\; & = & \;\int {\left(f_{\zeta }^{*}\right)}^{q}\;{\left(f_{\eta }^{*}\right)}^{1-q}\;d\mu \\ \; & = & \;\int \exp\left\{{\left(q\zeta \;+\;\left(1-q\right)\eta \right)}^{t}\;T\;-\;\left[q\kappa \left(\zeta \right)\;+\;\left(1-q\right)\kappa \left(\eta \right)\right]\right\}\;h\;d\mu \\ \; & = & \;\exp\left\{\kappa (q\zeta \;+\;(1-q)\eta )\;-\;[q\kappa (\zeta )\;+\;(1-q)\kappa (\eta )]\right\}\;, \end{array}$$

where the convexity of $\Xi^{*}$ ensures that κ(qζ+(1−q)η) is defined. □

**Remark** **2.** *For arbitrary divergence measures, several transformations and skewed versions as well as symmetrization methods, such as the Jensen–Shannon symmetrization, are studied in [19]. Applied to the KL-divergence, the skew Jensen–Shannon divergence is introduced as*

$$D_{JS_{q}}\left(P_{1},P_{2}\right)\;=\;q\;D_{KL}\left(P_{1},qP_{1}+\left(1-q\right)P_{2}\right)\;+\;\left(1-q\right)\;D_{KL}\left(P_{2},qP_{1}+\left(1-q\right)P_{2}\right)$$

*for $P_{1},P_{2}\in P$ and q∈(0,1), which includes the Jensen–Shannon distance for q=1/2 (the distance $D_{JS_{1/2}}^{1/2}$ even forms a metric). Note that, for $\zeta ,\eta \in \Xi^{*}$, the density $qf_{\zeta }^{*}+\left(1-q\right)f_{\eta }^{*}$ of the mixture $qP_{\zeta }^{*}+\left(1-q\right)P_{\eta }^{*}$ does not belong to P, in general, such that the identity in Theorem 1 for the KL-divergence is not applicable, here.*

*However, from the proof of Lemma 1, it is obvious that*

$$\frac{1}{A_{q}\left(\zeta ,\eta \right)}{\left(f_{\zeta }^{*}\right)}^{q}\;{\left(f_{\eta }^{*}\right)}^{1-q}\;=\;f_{q\zeta +(1-q)\eta }^{*}\;,\;\zeta ,\eta \in \Xi^{*}\;,\;q\in \left(0,1\right)\;,$$

*i.e., the EF P is closed when forming normalized weighted geometric means of the densities. This finding is utilized in [19] to introduce another version of the skew Jensen–Shannon divergence based on the KL-divergence, where the weighted arithmetic mean of the densities is replaced by the normalized weighted geometric mean. The skew geometric Jensen–Shannon divergence thus obtained is given by*

$$\begin{array}{c} D_{GJS_{q}}\left(\zeta ,\eta \right)\;=\;q\;D_{KL}\left(\zeta ,q\zeta +\left(1-q\right)\eta \right)\;+\;\left(1-q\right)\;D_{KL}\left(\eta ,q\zeta +\left(1-q\right)\eta \right)\;,\;\zeta ,\eta \in \Xi^{*}\;, \end{array}$$

*for q∈(0,1). By using Theorem 1, we find*

(13) $$\begin{array}{ccc} D_{GJS_{q}}\left(\zeta ,\eta \right)\; & = & \;q\;\left[\kappa \left(q\zeta +(1-q)\eta \right)-\kappa \left(\zeta \right)+\left(1-q\right)\;{\left(\zeta -\eta \right)}^{t}\pi \left(\zeta \right)\right]\\ & & \;+\;\left(1-q\right)\;\left[\kappa \left(q\zeta +(1-q)\eta \right)-\kappa \left(\eta \right)+q\;{\left(\eta -\zeta \right)}^{t}\pi \left(\eta \right)\right]\\ \; & = & \;\kappa \left(q\zeta +(1-q)\eta \right)\\ & & \;\;-\;\left[q\kappa (\zeta )+(1-q)\kappa (\eta )\right]\;+\;q\left(1-q\right)\;{\left(\zeta -\eta \right)}^{t}\left[\pi (\zeta )-\pi (\eta )\right]\\ \; & = & \;\ln\left(A_{q}\left(\zeta ,\eta \right)\right)\;+\;q\left(1-q\right)\;D_{J}\left(\zeta ,\eta \right)\;, \end{array}$$

*for $\zeta ,\eta \in \Xi^{*}$ and q∈(0,1).*

*In particular, setting q=1/2 gives the geometric Jensen–Shannon distance:*

$$D_{GJS}\left(\zeta ,\eta \right)\;=\;\kappa \left(\frac{\zeta +\eta }{2}\right)\;-\;\frac{\kappa (\zeta )+\kappa (\eta )}{2}\;+\;\frac{{\left(\zeta -\eta \right)}^{t}\left[\pi (\zeta )-\pi (\eta )\right]}{4}\;,\;\zeta ,\eta \in \Xi^{*}\;.$$

*For more details and properties as well as related divergence measures, we refer to [19,22].*

Formulas for $D_{R_{q}}$, $D_{CR_{q}}$, and $D_{H}$ are readily deduced from Lemma 1.

**Theorem** **2.** *Let P be as in Section 2 with minimal canonical representation (5). Then, for $\zeta ,\eta \in \Xi^{*}$ and q∈(0,1), we have*

$$\begin{array}{ccc} D_{R_{q}}\left(\zeta ,\eta \right)\; & = & \;\frac{1}{q(q-1)}\left[\kappa (q\zeta \;+\;(1-q)\eta )\;-\;[q\kappa (\zeta )\;+\;(1-q)\kappa (\eta )]\right]\;,\\ D_{CR_{q}}\left(\zeta ,\eta \right)\; & = & \;\frac{1}{q(q-1)}\left[\exp\left\{\kappa (q\zeta \;+\;(1-q)\eta )\;-\;[q\kappa (\zeta )\;+\;(1-q)\kappa (\eta )]\right\}\;-\;1\right]\;,\\ and\;D_{H}\left(\zeta ,\eta \right)\; & = & \;{\left(2\;-2\;\exp\left\{\kappa \left(\frac{\zeta \;+\;\eta }{2}\right)\;-\;\frac{\kappa (\zeta )\;+\;\kappa (\eta )}{2}\right\}\right)}^{1/2}\;. \end{array}$$

**Proof.**  Since

$$D_{R_{q}}\;=\;\frac{\ln(A_{q})}{q(q-1)}\;,\;D_{CR_{q}}\;=\;\frac{A_{q}-1}{q(q-1)}\;,\;\mathrm{and}\;D_{H}\;=\;{\left(2-2A_{1/2}\right)}^{1/2}\;,$$

the assertions are directly obtained from Lemma 1. □

It is well-known that

$$\lim_{q\to 1}D_{R_{q}}\left(P_{1},P_{2}\right)\;=\;D_{KL}\left(P_{1},P_{2}\right)\;\mathrm{and}\;\lim_{q\to 0}D_{R_{q}}\left(P_{1},P_{2}\right)\;=\;D_{KL}\left(P_{2},P_{1}\right)\;,$$

such that Formula (9) results from the representation of the Rényi divergence in Theorem 2 by sending *q* to 1.

The Sharma–Mittal divergence (see [1]) is closely related to the Rényi divergence as well and, by Theorem 2, a representation in EFs is available.

Moreover, representations within EFs for so-called local divergences can be derived as, e.g., the Cressie–Read local divergence, which results from the CR-divergence by multiplying the integrand with some kernel density function; see [23].

**Remark** **3.** *Inspecting the proof of Theorem 2, $D_{R_{q}}$ and $D_{CR_{q}}$ are seen to be strictly decreasing functions of $A_{q}$ for q∈(0,1); for q=1/2, this is also true for $D_{H}$. From an inferential point of view, this finding yields that, for fixed q∈(0,1), test statistics and pivot statistics based on these divergence measures will lead to the same test and confidence region, respectively. This is not the case within some divergence families such as $D_{R_{q}}$, q∈(0,1), where different values of q correspond to different tests and confidence regions, in general.*

A more general form of the Hellinger metric is given by

$$D_{H,m}\left(P_{1},P_{2}\right)\;=\;{\left(\int |f_{1}^{1/m}-f_{2}^{1/m}{|}^{m}\;d\mu \right)}^{1/m}$$

for m∈N, where $D_{H,2}=D_{H}$; see Formula (8). For m∈2N, i.e., if *m* is even, the binomial theorem then yields

[DH,m(P1,P2)]m=∫f11/m−f21/mmdμ=∑k=0m(−1)kmk∫f1k/mf2(m−k)/mdμ=∑k=0m(−1)kmkAk/m(P1,P2),

and inserting for $A_{k/m}$, k=1,1,…,m−1, according to Lemma 1 along with $A_{0}\equiv 1\equiv A_{1}$ gives a formula for $D_{H,m}$ in terms of the cumulant function of the EF P in Section 2. This representation is stated in [16].

Note that the representation for $A_{q}$ in Lemma 1 (and thus the formulas for $D_{R_{q}}$ and $D_{CR_{q}}$ in Theorem 2) are also valid for $\zeta ,\eta \in \Xi^{*}$ and q∈R∖[0,1] as long as $q\zeta +\left(1-q\right)\eta \in \Xi^{*}$ is true. This can be used, e.g., to find formulas for $D_{CR_{2}}$ and $D_{CR_{-1}}$, which coincide with the Pearson $\chi^{2}$-divergence

$$\begin{array}{ccc} D_{\chi^{2}}\left(\zeta ,\eta \right)\; & = & \;\frac{1}{2}\int \frac{{\left(f_{\zeta }^{*}-f_{\eta }^{*}\right)}^{2}}{f_{\eta }^{*}}\;d\mu \;=\;\frac{1}{2}\left[A_{2}\left(\zeta ,\eta \right)-1\right]\\ \; & = & \;\frac{1}{2}\left[\exp\left\{\kappa \left(2\zeta -\eta \right)\;-\;2\kappa \left(\zeta \right)+\kappa \left(\eta \right)\right\}-1\right] \end{array}$$

for $\zeta ,\eta \in \Xi^{*}$ with $2\zeta -\eta \in \Xi^{*}$ and the reverse Pearson $\chi^{2}$-divergence (or Neyman $\chi^{2}$-divergence) $D_{\chi^{2}}^{*}\left(\zeta ,\eta \right)=D_{\chi^{2}}\left(\eta ,\zeta \right)$ for $\zeta ,\eta \in \Xi^{*}$ with $2\eta -\zeta \in \Xi^{*}$. Here, the restrictions on the parameters are obsolete if $\Xi^{*}=R^{k}$ for some k∈N, which is the case for the EF of Poisson distributions and for any EF of discrete distributions with finite support such as binomial or multinomial distributions (with n∈N fixed). Moreover, quantities similar to $A_{q}$ such as $\int f_{\zeta }^{*}{\left(f_{\eta }^{*}\right)}^{\gamma }d\mu$ for γ>0 arise in the so-called γ-divergence, for which some representations can also be obtained; see [24] (Section 4).

**Remark** **4.** *If the assumption of the EF P to be regular is weakened to P being steep, Lemma 1 and Theorem 2 remain true; moreover, the formulas in Theorem 1 are valid for ζ lying in the interior of $\Xi^{*}$. Steep EFs are full EFs in which boundary points of $\Xi^{*}$ that belong to $\Xi^{*}$ satisfy a certain property. A prominent example is provided by the full EF of inverse normal distributions. For details, see, e.g., [13].*

The quantity $A_{q}$ in Formula (12) is the two-dimensional case of the weighted Matusita affinity

(14) $$\rho_{w_{1},\ldots ,w_{n}}\left(P_{1},\ldots ,P_{n}\right)\;=\;\int {\left(\prod_{i=1}^{n}f_{i}\right)}^{w_{i}}\;d\mu$$

for distributions $P_{1},\ldots ,P_{n}$ with μ-densities $f_{1},\ldots ,f_{n}$, weights $w_{1},\ldots ,w_{n}>0$ satisfying $\sum_{i=1}^{n}w_{i}=1$, and n≥2; see [4] (p. 49) and [6]. $\rho_{w_{1},\ldots ,w_{n}}$, in turn, is a generalization of the Matusita affinity

$$\rho_{n}\left(P_{1},\ldots ,P_{n}\right)=\int {\left(\prod_{i=1}^{n}f_{i}\right)}^{1/n}\;d\mu$$

introduced in [25,26]. Along the lines of the proof of Lemma 1, we find the representation

$$\rho_{w_{1},\ldots ,w_{n}}\left(\zeta^{\left(1\right)},\ldots ,\zeta^{\left(n\right)}\right)\;=\;\exp\left\{\kappa \left(\sum_{i=1}^{n}w_{i}\zeta^{\left(i\right)}\right)-\sum_{i=1}^{n}w_{i}\kappa \left(\zeta^{\left(i\right)}\right)\right\}\;,\;\zeta^{\left(1\right)},\ldots ,\zeta^{\left(n\right)}\in \Xi^{*}\;,$$

for the EF P in Section 2; cf. [27]. In [4], the quantity in Formula (14) is termed Hellinger transform, and a representation within EFs is stated in Example 1.88.

$\rho_{w_{1},\ldots ,w_{n}}$ can be used, for instance, as the basis of a homogeneity test (with null hypothesis $H_{0}:\zeta^{\left(1\right)}=\ldots =\zeta^{\left(n\right)}$) or in discriminant problems.

For a representation of an extension of the Jeffrey distance to more than two distributions in an EF, the so-called Toussaint divergence, along with statistical applications, we refer to [8].

## 4. Entropy Measures

The literature on entropy measures, their applications, and their relations to divergence measures is broad. We focus on some selected results and state several simple representations of entropy measures within EFs.

Let the EF in Section 2 be given with h≡1, which is the case, e.g., for the one-parameter EFs of geometric distributions and exponential distributions as well as for the two-parameter EF of univariate normal distributions. Formula (5) then yields that

$$\int {\left(f_{\zeta }^{*}\right)}^{r}\;d\mu \;=\;\int e^{r\zeta^{t}T-r\kappa \left(\zeta \right)}\;d\mu \;=\;e^{\kappa (r\zeta )-r\kappa (\zeta )}\;=\;J_{r}\left(\zeta \right)\;,\;\mathrm{say},$$

for r>0 and $\zeta \in \Xi^{*}$ with $r\zeta \in \Xi^{*}$. Note that the latter condition is not that restrictive, since the natural parameter space of a regular EF is usually a cartesian product of the form $A_{1}\times \ldots \times A_{k}$ with $A_{i}\in \left\{R,\left(-\infty ,0\right),\left(0,\infty \right)\right\}$ for 1≤i≤k.

The Taneja entropy is then obtained as

$$\begin{array}{ccc} H_{T}\left(\zeta \right)\; & = & \;-\;2^{r-1}\int {\left(f_{\zeta }^{*}\right)}^{r}\;\ln\left(f_{\zeta }^{*}\right)\;d\mu \;=\;-\;2^{r-1}\left(\zeta^{t}\int T\;e^{r\zeta^{t}T-r\kappa \left(\zeta \right)}\;d\mu \;-\;\kappa \left(\zeta \right)\;J_{r}\left(\zeta \right)\right)\\ \; & = & \;-\;2^{r-1}\;J_{r}\left(\zeta \right)\;\left(\zeta^{t}\int T\;f_{r\zeta }^{*}\;d\mu \;-\;\kappa \left(\zeta \right)\right)\;=\;-2^{r-1}\;e^{\kappa (r\zeta )-r\kappa (\zeta )}\;\left(\zeta^{t}\pi \left(r\zeta \right)-\kappa \left(\zeta \right)\right) \end{array}$$

for r>0 and $\zeta \in \Xi^{*}$ with $r\zeta \in \Xi^{*}$, which includes the Shannon entropy

$$H_{S}\left(\zeta \right)\;=\;-\int f_{\zeta }^{*}\;\ln\left(f_{\zeta }^{*}\right)\;d\mu \;=\;\kappa \left(\zeta \right)-\zeta^{t}\pi \left(\zeta \right)\;,\;\zeta \in \Xi^{*}\;,$$

by setting r=1; see [7,28].

Several other important entropy measures are functions of $J_{r}$ and therefore admit respective representations in terms of the cumulant function of the EF. Two examples are provided by the Rényi entropy and the Havrda–Charvát entropy (or Tsallis entropy), which are given by

$$\begin{array}{ccc} H_{R_{r}}\left(\zeta \right)\; & = & \;\frac{1}{1-r}\ln\left(J_{r}\left(\zeta \right)\right)\;=\;\frac{\kappa (r\zeta )-r\kappa (\zeta )}{1-r}\;,\;r>0\;,r\neq 1\;,\\ \mathrm{and}\;H_{HC_{r}}\left(\zeta \right)\; & = & \;\frac{1}{1-r}\left(J_{r}\left(\zeta \right)-1\right)\;=\;\frac{1}{1-r}\left(e^{\kappa (r\zeta )-r\kappa (\zeta )}-1\right)\;,\;r>0,\;r\neq 1\;, \end{array}$$

for $\zeta \in \Xi^{*}$ with $r\zeta \in \Xi^{*}$; for the definitions, see, e.g., [1]. More generally, the Sharma–Mittal entropy is seen to be

$$\begin{array}{ccc} H_{SM_{r,s}}\left(\zeta \right)\; & = & \;\frac{1}{1-s}\left[{\left(J_{r}\left(\zeta \right)\right)}^{\frac{1-s}{1-r}}-1\right]\\ \; & = & \;\frac{1}{1-s}\left[{\left(e^{\kappa (r\zeta )-r\kappa (\zeta )}\right)}^{\frac{1-s}{1-r}}-1\right]\;,\;r>0\;,r\neq 1\;,\;s\in R\;,s\neq 1\;, \end{array}$$

for $\zeta \in \Xi^{*}$ with $r\zeta \in \Xi^{*}$, which yields the representation for $H_{S}$ as r=s→1, for $H_{R_{r}}$ as s→1, and for $H_{HC_{r}}$ as s→r; see [29].

If the assumption h≡1 is not met, the calculus of the entropies becomes more involved. The Shannon entropy, for instance, is then given by

$$H_{S}\left(\zeta \right)\;=\;\kappa \left(\zeta \right)-\zeta^{t}\pi \left(\zeta \right)+E_{\zeta }\left[\ln\left(h\right)\right]\;,\;\zeta \in \Xi^{*}\;,$$

where the additional additive term $E_{\zeta }\left[\ln\left(h\right)\right]$, as it is the mean of ln(h) under $P_{\zeta }^{*}$, will also depend on ζ, in general; see, e.g., [17]. Since

$$\int {\left(f_{\zeta }^{*}\right)}^{r}\;d\mu \;=\;e^{\kappa (r\zeta )-r\kappa (\zeta )}\;E_{r\zeta }\left[h^{r-1}\right]$$

for r>0 and $\zeta \in \Xi^{*}$ with $r\zeta \in \Xi^{*}$ (cf. [29]), more complicated expressions result for other entropies and require to compute respective moments of *h*. Of course, we arrive at the same expressions as for the case h≡1 if the entropies are introduced with respect to the dominating measure ν, which is neither a counting nor a Lebesgue measure, in general; see Section 2. However, in contrast to divergence measures, entropies usually depend on the dominating measure, such that the resulting entropy values of the distributions will be different.

Representations of Rényi and Shannon entropies for various multivariate distributions including several EFs can be found in [30].

## 5. Application

As aforementioned, applications of divergence measures in statistical inference have been extensively discussed; see the references in the introduction. As an example, we make use of the representations of the symmetric divergences (distances) in Section 3 to construct confidence regions that are different from the standard rectangles for exponential parameters in a multi-sample situation.

Let $n_{1},\ldots ,n_{k}\in N$ and $X_{ij}$, 1≤i≤k, $1\leq j\leq n_{i}$, be independent random variables, where $X_{i1},\ldots ,X_{in_{i}}$ follow an exponential distribution with (unknown) mean $1/\alpha_{i}$ for 1≤i≤k. The overall joint distribution $P_{\alpha }$, say, has the density function

(15) $$f_{\alpha }\left(x\right)\;=\;e^{\alpha^{t}T\left(x\right)-\kappa \left(\alpha \right)}\;,$$

with the *k*-dimensional statistic

$$T\left(x\right)\;=\;-\;{\left(x_{1•},\ldots ,x_{k•}\right)}^{t}\;,\;\mathrm{where}\;x_{i•}=\sum_{j=1}^{n_{i}}x_{ij}\;,\;1\leq i\leq k\;,$$

for $x=\left(x_{11},\ldots ,x_{1n_{1}},\ldots ,x_{k1},\ldots ,x_{kn_{k}}\right)\in {\left(0,\infty \right)}^{n}$, the cumulant function

$$\kappa \left(\alpha \right)\;=\;-\sum_{i=1}^{k}n_{i}\ln\left(\alpha_{i}\right)\;,\;\alpha ={\left(\alpha_{1},\ldots ,\alpha_{k}\right)}^{t}\in {\left(0,\infty \right)}^{k}\;,$$

and $n=\sum_{i=1}^{k}n_{i}$. It is easily verified that $P=\{P_{\alpha }:\alpha \in {\left(0,\infty \right)}^{k}\}$ forms a regular EF with minimal canonical representation (15). The corresponding mean value function is given by

$$\pi \left(\alpha \right)\;=\;-{\left(\frac{n_{1}}{\alpha_{1}},\ldots ,\frac{n_{k}}{\alpha_{k}}\right)}^{t}\;,\;\alpha ={\left(\alpha_{1},\ldots ,\alpha_{k}\right)}^{t}\in {\left(0,\infty \right)}^{k}\;.$$

To construct confidence regions for α based on the Jeffrey distance $D_{J}$, the resistor-average distance $D_{RA}$, the distance $D_{GA}$, the Hellinger metric $D_{H}$, and the geometric Jensen–Shannon distance $D_{GJS}$, we first compute the KL-divergence $D_{KL}$ and the affinity $A_{1/2}$. Note that, by Remark 3, constructing a confidence region based on $D_{H}$ is equivalent to constructing a confidence region based on either $A_{1/2}$, $D_{R_{1/2}}$, or $D_{CR_{1/2}}$.

For $\alpha ={\left(\alpha_{1},\ldots ,\alpha_{k}\right)}^{t},\beta ={\left(\beta_{1},\ldots ,\beta_{k}\right)}^{t}\in {\left(0,\infty \right)}^{k}$, we obtain from Theorem 1 that

$$\begin{array}{ccc} D_{KL}\left(\alpha ,\beta \right)\; & = & \;-\sum_{i=1}^{k}n_{i}\ln\left(\beta_{i}\right)+\sum_{i=1}^{k}n_{i}\ln\left(\alpha_{i}\right)-\sum_{i=1}^{k}\frac{n_{i}}{\alpha_{i}}\left(\alpha_{i}-\beta_{i}\right)\\ \; & = & \;\sum_{i=1}^{k}n_{i}\left[\frac{\beta_{i}}{\alpha_{i}}-\ln\left(\frac{\beta_{i}}{\alpha_{i}}\right)-1\right]\;, \end{array}$$

such that

$$\begin{array}{ccc} D_{J}\left(\alpha ,\beta \right)\; & = & \;D_{KL}\left(\alpha ,\beta \right)+D_{KL}\left(\beta ,\alpha \right)\\ \; & = & \;\sum_{i=1}^{k}n_{i}\left(\frac{\alpha_{i}}{\beta_{i}}+\frac{\beta_{i}}{\alpha_{i}}-2\right)\;. \end{array}$$

$D_{RA}$ and $D_{GA}$ are then computed by inserting for $D_{KL}$ and $D_{J}$ in Formulas (10) and (11). Applying Lemma 1 yields

$$\begin{array}{ccc} A_{1/2}\left(\alpha ,\beta \right)\; & = & \;\left[\prod_{i=1}^{k}{\left(\frac{\alpha_{i}+\beta_{i}}{2}\right)}^{-n_{i}}\right]\;\left(\prod_{i=1}^{k}\alpha_{i}^{n_{i}/2}\right)\;\left(\prod_{i=1}^{k}\beta_{i}^{n_{i}/2}\right)\\ \; & = & \;\prod_{i=1}^{k}{\left[\frac{1}{2}\left(\sqrt{\frac{\alpha_{i}}{\beta_{i}}}+\sqrt{\frac{\beta_{i}}{\alpha_{i}}}\right)\right]}^{-n_{i}}\;, \end{array}$$

which gives $D_{H}\left(\alpha ,\beta \right)={\left[2-2A_{1/2}\left(\alpha ,\beta \right)\right]}^{1/2}$ by inserting, and, by using Formula (13), also leads to

$$\begin{array}{ccc} D_{GJS}\left(\alpha ,\beta \right)\; & = & \;\ln\left(A_{1/2}\left(\alpha ,\beta \right)\right)+\frac{D_{J}\left(\alpha ,\beta \right)}{4}\\ \; & = & \;\frac{1}{4}\sum_{i=1}^{k}n_{i}\left[\frac{\alpha_{i}}{\beta_{i}}+\frac{\beta_{i}}{\alpha_{i}}-4\ln\left(\frac{1}{2}\left(\sqrt{\frac{\alpha_{i}}{\beta_{i}}}+\sqrt{\frac{\beta_{i}}{\alpha_{i}}}\right)\right)-2\right]\;. \end{array}$$

The MLE $\hat{\alpha }={\left({\hat{\alpha }}_{1},\ldots ,{\hat{\alpha }}_{k}\right)}^{t}$ of α based on $X=(X_{11},\ldots ,X_{1n_{1}},\ldots ,X_{k1},\ldots ,X_{kn_{k}})$, is given by

$$\hat{\alpha }\;=\;{\left(\frac{n_{1}}{X_{1•}},\ldots ,\frac{n_{k}}{X_{k•}}\right)}^{t}\;,$$

where ${\hat{\alpha }}_{1},\ldots ,{\hat{\alpha }}_{k}$ are independent. By inserting the random distances $D_{J}\left(\hat{\alpha },\alpha \right)$, $D_{RA}\left(\hat{\alpha },\alpha \right)$, $D_{GA}\left(\hat{\alpha },\alpha \right)$, $D_{H}\left(\hat{\alpha },\alpha \right)$, and $D_{GJS}\left(\hat{\alpha },\alpha \right)$ turn out to depend on X only through the vector ${\left(\alpha_{1}/{\hat{\alpha }}_{1},\ldots ,\alpha_{k}/{\hat{\alpha }}_{k}\right)}^{t}$ of component-wise ratios, where $\alpha_{i}/{\hat{\alpha }}_{i}$ has a gamma distribution with shape parameter $n_{i}$, scale parameter $1/n_{i}$, and mean 1 for 1≤i≤k. Since these ratios are moreover independent, the above random distances form pivot statistics with distributions free of α.

Now, confidence regions for α with confidence level p∈(0,1) are given by

$$\begin{array}{ccc} C_{•}\; & = & \;\left\{\alpha \in {\left(0,\infty \right)}^{k}:\;D_{•}\left(\hat{\alpha },\alpha \right)\leq c_{•}\left(p\right)\right\}\;, \end{array}$$

where $c_{•}\left(p\right)$ denotes the *p*-quantile of $D_{•}\left(\hat{\alpha },\alpha \right)$ for •=J,RA,GA,H,GJS, numerical values of which can readily be obtained via Monte Carlo simulation by sampling from gamma distributions.

Confidence regions for the mean vector $m={\left(1/\alpha_{1},\ldots ,1/\alpha_{k}\right)}^{t}$ with confidence level p∈(0,1) are then given by

$$\begin{array}{ccc} {\tilde{C}}_{•}\; & = & \;\left\{{\left(\frac{1}{\alpha_{1}},\ldots ,\frac{1}{\alpha_{k}}\right)}^{t}\in {\left(0,\infty \right)}^{k}:\;{\left(\alpha_{1},\ldots ,\alpha_{k}\right)}^{t}\in C_{•}\right\} \end{array}$$

for •=J,RA,GA,H,GJS.

**Figure 1:**
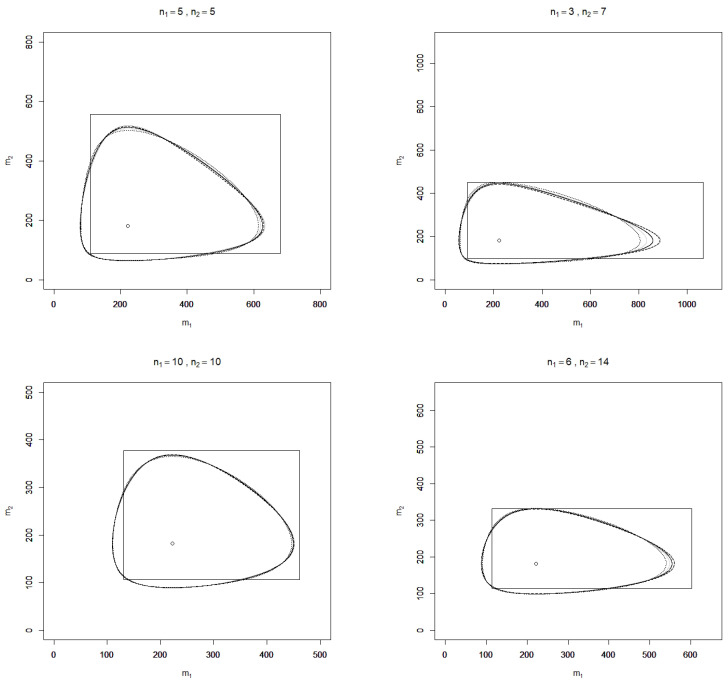
Illustration of the confidence regions ${\tilde{C}}_{J}$ (solid light grey line), ${\tilde{C}}_{RA}$ (solid dark grey line), ${\tilde{C}}_{GA}$ (solid black line), ${\tilde{C}}_{H}$ (dashed black line), ${\tilde{C}}_{GJS}$ (dotted black line), and *R* (rectangle) for the mean vector $m={\left(m_{1},m_{2}\right)}^{t}$ with level 90% and sample sizes $n_{1},n_{2}$ based on a realization $\hat{\alpha }={\left(0.0045,0.0055\right)}^{t}$, respectively $\hat{m}={\left(222.2,181.8\right)}^{t}$ of the MLE (circle).

**Figure 2:**
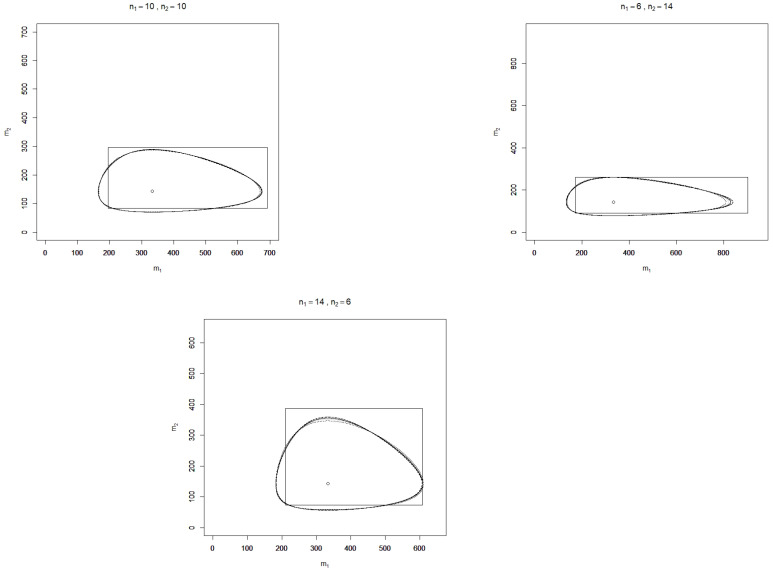
Illustration of the confidence regions ${\tilde{C}}_{J}$ (solid light grey line), ${\tilde{C}}_{RA}$ (solid dark grey line), ${\tilde{C}}_{GA}$ (solid black line), ${\tilde{C}}_{H}$ (dashed black line), ${\tilde{C}}_{GJS}$ (dotted black line), and *R* (rectangle) for the mean vector $m={\left(m_{1},m_{2}\right)}^{t}$ with level 90% and sample sizes $n_{1},n_{2}$ based on a realization $\hat{\alpha }={\left(0.003,0.007\right)}^{t}$, respectively $\hat{m}={\left(333.3,142.9\right)}^{t}$ of the MLE (circle).

In Figure 1 and Figure 2, realizations of ${\tilde{C}}_{J}$, ${\tilde{C}}_{RA}$, ${\tilde{C}}_{GA}$, ${\tilde{C}}_{H}$, and ${\tilde{C}}_{GJS}$ are depicted for the two-sample case (k=2) and some sample sizes $n_{1},n_{2}$ and values of $\hat{\alpha }={\left({\hat{\alpha }}_{1},{\hat{\alpha }}_{2}\right)}^{t}$, where the confidence level is chosen as p=90%. Additionally, realizations of the standard confidence region

$$R\;=\;\left[\frac{2n_{1}}{{\hat{\alpha }}_{1}\chi_{1-q}^{2}\left(2n_{1}\right)}\;,\;\frac{2n_{1}}{{\hat{\alpha }}_{1}\chi_{q}^{2}\left(2n_{1}\right)}\right]\times \left[\frac{2n_{2}}{{\hat{\alpha }}_{2}\chi_{1-q}^{2}\left(2n_{2}\right)}\;,\;\frac{2n_{2}}{{\hat{\alpha }}_{2}\chi_{q}^{2}\left(2n_{2}\right)}\right]$$

with a confidence level of 90% for $m={\left(m_{1},m_{2}\right)}^{t}$ are shown in the figures, where $q=(1-\sqrt{0.9})/2$ and $\chi_{\gamma }^{2}\left(v\right)$ denotes the γ-quantile of the chi-square distribution with *v* degrees of freedom.

It is found that over the sample sizes and realizations of $\hat{\alpha }$ considered, the confidence regions ${\tilde{C}}_{J}$, ${\tilde{C}}_{RA}$, ${\tilde{C}}_{GA}$, ${\tilde{C}}_{H}$, and ${\tilde{C}}_{GJS}$ are similarly shaped but do not coincide as the plots for different sample sizes show. In terms of (observed) area, all divergence-based confidence regions perform considerably better than the standard rectangle. This finding, however, depends on the parameter of interest, which here is the vector of exponential means; for the divergence-based confidence regions and the standard rectangle for α itself, the contrary assertion is true. Although the divergence-based confidence regions have a smaller area than the standard rectangle, this is not at the cost of large projection lengths with respect to the $m_{1}$- and $m_{2}$-axes, which serve as further characteristics for comparing confidence regions. Monte Carlo simulations may moreover be applied to compute the expected area and projection lengths as well as the coverage probabilities of false parameters for a more rigorous comparison of the performance of the confidence regions, which is beyond the scope of this article.

## Abbreviations

CR	Cressie–Read
EF	exponential family
KL	Kullback–Leibler
MLE	maximum likelihood estimator
